# Access to care of frail community-dwelling older adults in Belgium: a qualitative study

**DOI:** 10.1017/S1463423619000100

**Published:** 2019-06-07

**Authors:** Bram Fret, Liesbeth De Donder, Deborah Lambotte, Sarah Dury, Michaël Van der Elst, Nico De Witte, Lise Switsers, Sylvia Hoens, Sofie Van Regenmortel, Dominique Verté

**Affiliations:** 1 Doctor in Educational Sciences, Department of Educational Sciences, Vrije Universiteit Brussel, Brussels, Belgium; 2 Professor, Department of Educational Sciences, Vrije Universiteit Brussel, Brussels, Belgium; 3 Postdoctoral Researcher, Research Foundation Flanders (FWO), Brussels, Belgium; 4 Doctoral Researcher, Department of Public Health and Primary Care, Katholieke Universiteit Leuven, Leuven, Belgium; 5 Lecturer, Faculty Education, Health and Social Work, Hogeschool Gent, Ghent, Belgium; 6 Doctoral Researcher, Department of Educational Sciences, Vrije Universiteit Brussel, Brussels, Belgium; 7 Doctoral Researcher, Research Foundation Flanders (FWO), Brussels, Belgium

**Keywords:** accessibility, care and support, elderly, frailty, qualitative research

## Abstract

**Aim:**

This paper aims to identify barriers that frail community-dwelling older adults experience regarding access to formal care and support services.

**Background:**

Universal access to healthcare has been set by the World Health Organisation (WHO) as a main goal for the post-2015 development agenda. Nevertheless, regarding access to care, particular attention has to be paid to the so-called vulnerable groups, such as (frail) older adults.

**Methods:**

Both inductive and deductive content analyses were performed on 22 individual interviews with frail, community-dwelling older adults who indicated they lacked care and support. The coding scheme was generated from the conceptual framework ‘6A’s of access to care and support’ (referring to work of Penchansky and Thomas, 1981; Wyszewianski, 2002; Saurman, 2016) and applied on the transcripts.

**Findings:**

Results indicate that (despite all policy measures) access to a broad spectrum of care and support services remains a challenge for older people in Belgium. The respondents’ barriers concern: ‘affordability’ referring to a lot of Belgian older adults having limited pensions, ‘accessibility’ going beyond geographical accessibility but also concerning waiting lists, ‘availability’ referring to the lack of having someone around, ‘adequacy’ addressing the insufficiency of motivated staff, the absence of trust in care providers influencing ‘acceptability’, and ‘awareness’ referring to limited health literacy. The discussion develops the argument that in order to make care and support more accessible for people in order to be able to age in place, governments should take measures to overcome these access limitations (eg, by automatic entitlements) and should take into account a broad description of access. Also, a seventh barrier (a seventh A) within the results, namely ‘ageism’, was discovered.

## Introduction

The World Health Organisation (WHO) pointed out universal access (ie, the absence of sociocultural, organisational, economic, geographical and gender-related barriers) to healthcare as an overarching goal for health in the post-2015 development agenda (Evans, Hsu and Boerma, 2013; Marziale, 2016). This is recognised by the United Nations sustainable development goals by which all of its United Nations member states have agreed to try to achieve universal health coverage (ie, the capacity of health systems to respond to the populations’ needs at any care level, without causing financial damage) by 2030 (World Health Organisation, 2018a). Universal health coverage includes financial risk protection, access to quality essential healthcare services and access to safe, effective, quality and affordable essential medicines and vaccines for all (World Health Organisation, 2018b). Regarding health, and particularly access to healthcare, attention must be paid not only to the so-called vulnerable groups, such as homeless people, newly arrived immigrants, sex workers, or drug users, but also to frail older adults (Rijksinstituut voor Ziekte-en Invaliditeitsverzekering, 2014; Rowe, Fulmer and Fried, 2016). Frailty is a common phenomenon in community-dwelling older adults that is often used in research as a (clinical) phenotype (Fried *et al*., 2001) or an accumulation of health deficits (Rockwood *et al*., 1994; Etman *et al*., 2012). More recently, multidimensional approaches have defined frailty as ‘a dynamic state that affects an individual who experiences losses in one or more domains (physical, psychological, social, and more recently, also environmental)’ (De Witte *et al.*, 2013 a and b). Also different researchers point to the necessity to operationalise frailty as a multidimensional and dynamic concept that considers the complex interplay of physical, cognitive, psychological, social and environmental factors (Bergman *et al.*, 2007; Armstrong *et al*., 2010; De Witte *et al.*, 2013 a and b). The word frailty has a stigma attached pointing towards losses and decline. However, frailty has not solely negative consequences in daily life, especially when the right care and support is present. Besides measuring the deficits of frailty, there is also a need to take into account the strengths and resources of older adults (Buntinx *et al*., 2004). Moreover, in the Dutch language, the word ‘frailty’ is translated as ‘kwetsbaarheid’, which has not got such a negative connotation and stigma. This paper aims to identify barriers that frail community-dwelling older adults experience regarding access to formal care and support.

Research on access to health services appears particularly important with the rising proportion of older adults. International research often associates barriers affecting access to healthcare for older adults with the lack of health insurance (Fitzpatrick *et al*., 2004; Thorpe *et al*., 2011) or is about specific populations and conditions (eg, dental care, people facing chronic conditions, people living in rural areas, etc.) (White *et al*., 2002; Goins *et al*., 2005; Wallace and Guitérrez, 2005). In Belgium, insurance status is a minor problem, because health insurance is nationally organised and compulsory. Everyone living and/or working in Belgium is required to take an insurance in the event of illness or indemnity through membership of a health insurance fund (Belgium.be, 2018). Care policy in Belgium is both a responsibility of the federal authorities and federated entities (regions and communities). The federal authorities are mainly responsible for the regulation and financing of the compulsory health insurance, while the federated entities are in charge of health promotion and prevention, different aspects of community care and support services (family aids, cleaning aids, meals on wheels, etc.) and the coordination and collaboration in primary healthcare and palliative care. To facilitate cooperation between the federal authorities and the federated entities, inter-ministerial conferences are regularly organised (Gerkens and Merkur, 2010; Dumont, 2015). Nevertheless, several challenges in terms of access to care and support in Belgium remain. While the average level of unmet care needs is rather low (0.1% for high incomes and 5.5% among low incomes in 2013) for Belgian inhabitants, the Organisation for Economic Cooperation and Development (OECD, 2016) states that Belgium shows large inequalities: low-income people more often forgo health examinations due to costs, travelling distance or waiting time, compared with high-income people. Despite universal coverage, on average, 8% of Belgian households declared that in 2013 they had to postpone healthcare for financial reasons (eg, medical care, surgery, dental care, prescribed medicines, mental healthcare, eyeglasses or contact lenses). Moreover, the share of out-of-pocket payments (ie, expenditures covered directly by the patient because healthcare insurance does not cover the full amount) is relatively high in Belgium compared with other European countries (18% of total health expenditures). Among older adults, special attention should be drawn to the accessibility and sustainability of long-term care services (Vrijens *et al*., 2015).

Access to care, however, is more than being able to pay for care or support expenditures. Already more than 30 years ago, Penchansky and Thomas (1981) published an article in this area entitled, ‘The concept of access: Definition and Relation to Consumer Satisfaction’. Nevertheless, this framework is still commonly used, not only concerning access to healthcare (Clark and Coffee, 2011; Derose, Gresenz and Ringel, 2011; Levesque, Harris and Russell, 2013) but also in a broader context of access to services (United Nations Educational, Scientific and Cultural Organisation, 2013), for example to discover access barriers to healthy food (Usher, 2015; Zhang, 2017), access to energy security (Cherp and Jewell, 2014) and access to education (Lee, 2016). Also, the recent research of Saurman (2016) has re-evaluated, improved and extended Penchansky and Thomas’ framework. Penchansky and Thomas (1981: 1) describe access as ‘a general concept that summarises a set of more specific dimensions describing the fit between the patient and the healthcare system’. These specific dimensions are the five As [affordability, availability, accessibility, adequacy (or accommodation) and acceptability] of access to care. As the framework already dates from 1981, the definition given to the five As seems dated and complex. In a more recent editorial column titled, ‘Access to Care: Remembering Old Lessons’, Wyszewianski (2002: 1441) gave an updated description connecting with the (then) current society. He defines the five As of access as follows:

1. ‘*Affordability* is determined by how the provider’s charges relate to the client’s ability and willingness to pay for services’;
2. ‘*Availability* measures the extent to which the provider has the requisite resources, such as personnel and technology, to meet the needs of the client’;
3. ‘*Accessibility* refers to geographic accessibility, which is determined by how easily the client can physically reach the provider’s location’;
4. ‘*Adequacy* (*or accommodation*) reflects the extent to which the provider’s operation is organised in ways that meet the constraints and preferences of the client. Of greatest concern are hours of operation, how telephone communications are handled and the client’s ability to receive care without prior appointments’;
5. ‘*Acceptability* captures the extent to which the client is comfortable with the more immutable characteristics of the provider, and vice versa. These characteristics include the age, sex, social class, and ethnicity of the provider (and of the client), as well as the diagnosis and type of coverage of the client’.

Recently, Saurman (2016: 37) proposed a sixth dimension to further develop the framework of access of Penchansky and Thomas, namely awareness:

‘*Awareness* refers to effective communication and information strategies with relevant users (clinicians, patients, the broader community)’.

Saurman links the concept of awareness to the challenge of health literacy. ‘Health literacy’ is defined as the ‘degree to which individuals have the capacity to obtain, process, and understand basic health information and services needed to make appropriate health decisions’ (Parker and Ratzan, 2010: 20). Low literacy may cause health disparities, especially among older adults inadequate health literacy is associated with poorer physical and mental health (Wolf, Gazmararian and Baker, 2005; Saha, 2006; Chesser *et al*., 2016). Recent studies also revealed that advanced age might result in a significant increase in the prevalence of inadequate health literacy which demands a tailored approach (Zamora and Clingerman, 2011; Manofo and Wong, 2012).

In this study, we focus on one of the above defined vulnerable groups deserving special attention, namely community-dwelling older adults. Despite being a major policy goal, the challenge of access to care among community-dwelling older adults is still under-researched, especially using a structured framework (Evans, Hsu and Boerma, 2013). As older people are major consumers of healthcare, the growing proportion of older people in European populations does present some challenges concerning their access to the healthcare and welfare system as well to the affordability for providing institutions (World Health Organisation, 2014).

Facing the mentioned research gaps, this research is handling challenges of general access to care and support of frail, community-dwelling older adults using a broad and comprehensive framework. In doing so, the following central research question is addressed: which barriers do frail, community-dwelling older adults experience to access formal care and support services? To detect these barriers, we use the five As of access to care from Penchansky and Thomas (1981) as they are described by Wyszewianski (2002) and the sixth A (awareness) as added by Saurman (2016) together resulting in a new framework of ‘six As of access to care and support’.

## Methods

### Data collection

For this paper, data collected within the Detection, Support and Care for older people – Prevention and Empowerment (D-SCOPE) project were used. The D-SCOPE project is a 4-year research project (2015–18) that investigates strategies for proactive detection of potentially frail, community-dwelling older people, in order to guide them towards adequate support and/or care with a focus on empowerment. The general aim of the second phase of the D-SCOPE-research, which this paper concerns, was to gain information concerning the experiences and understandings of older people concerning frailty and their possibility to age in place. The Ethical Commission Human Sciences of the Vrije Universiteit Brussel approved the study (file number ECHW_031). Older people were asked to sign an informed consent agreement. In case they were not capable of signing this document, a family member or another legal representative was allowed to sign it on their behalf, as stipulated by the Belgian civil code. Respondents were informed about the voluntary nature of their involvement in the study, their right to refuse to answer and the privacy of their responses. Also, respondents had the right not to participate in the study and to withdraw their consent at any time without negative consequences. Refusal to consent led to exclusion of the study.

The overall data collection within the second phase of the D-SCOPE research comprised data of 121 community-dwelling older adults (60+) in the Dutch-speaking part of Belgium and in Brussels. These interviews took place in participants’ homes or in the local service centre. Data were collected between November 2015 and March 2016. Respondents were purposively sampled based on risk profiles for multidimensional frailty, which included age, gender, marital status, level of education, household income, whether the respondent had moved in the previous 10 years and country of birth (Dury *et al*., 2016). Hospitalisation and any state that may interfere with a good understanding of the questions (being too sick to participate in the interview, etc.) (according to the participant or an informal caregiver) or also the inability to provide adequate answers during the face-to-face interviews (as noted by the interviewer) were exclusion criteria. The presence of dementia was also an exclusion criterion. The current paper reports on a selection of 22 face-to-face interviews.

### Interview scheme

Nine trained researchers conducted a quantitative questionnaire and a qualitative semi-structured interview. The quantitative questionnaire comprised questions related to sociodemographic and socioeconomic characteristics and the Comprehensive Frailty Assessment Instrument (CFAI) (De Witte *et al.*, 2013b), which is a self-administered instrument and measures four domains of frailty from a holistic approach. The CFAI contains 23 indicators and demonstrates a high overall internal consistency and high consistency of its scales, thus supporting the validity and reliability of the instrument and highlighting to the multidimensionality of frailty. The CFAI has been proven to be internally consistent, with a Cronbach’s *α* of 0.812 that explains 63.6% of the variance in frailty (De Witte *et al.*, 2013c). For the physical domain of frailty, the respondent’s general physical health was assessed using four items, such as whether they could walk up a hill or stairs. The psychological domain was captured by measuring mood-disorders and emotional loneliness (eight items, eg, feeling unhappy or depressed). The social domain of frailty was evaluated by older people’s social loneliness (three items, eg, ‘I feel an emptiness around me’) and their potential social support network (10 items, eg, partner, children, neighbours). Finally, environmental frailty was assessed by propositions regarding the suitability of the physical housing environment (five items, eg, the house is in a bad state). Cognitive frailty was originally not included in the original CFAI. Four questions were added to the CFAI to assess subjective cognitive frailty, which resulted in the CFAI-plus (keeping good psychometric qualities) (De Roeck *et al.*, 2018). Finally, the sufficiency of care and support was assessed with a one-item question, eg, ‘On a scale from 0 to 10, to what extent do you feel that the care and support you receive is sufficient?’. Scores ranged from 0 (bad) to 10 (excellent) on a numerical rating scale. To assess the significance of that score, each answer was followed by a question to indicate whether the participant perceived the score as poor, average or good.

After the quantitative part, the same researchers held a semi-structured face-to-face interview with open-ended questions with the participants. This was the main part of the second phase of the D-SCOPE research. The topic list consisted of four main questions: (a) ‘How do you experience frailty and what does frailty mean to you?’; (b) ‘How do you experience frailty has an effect on your quality of life, care and support, meaning in life, and to what extent do you still have control over the things happening in your life?’; (c) ‘What should an older person do, have or need to maintain his/her quality of life when becoming frail?’; (d) ‘What were the highlights and what were the low points in your life during the past year, did changes occur? And how do you feel about the future?’. The topic list was developed within the D-SCOPE research group, which consists of researchers specialised in gerontology and/or frailty and representing several disciplines (eg, old-age medicine, psychology, educational sciences, etc.). A panel of experts approved all questions, indicating for content validity in the interview (Landsheer and Boeije, 2010). The expert panel consisted of two neurologists specialised in dementia, a psychologist specialised in neuropsychology and dementia, five adult educational scientists specialised in social gerontology, three general practitioners specialised in frailty in later life and two social gerontologists specialised in public health. Researchers that conducted the interviews received training and several scenarios were developed in order to address potential difficulties (eg, difficulties in understanding the concept frailty) (Dury *et al.*, 2018). All researchers also received a list of definitions explaining the terms used in the questionnaire. This list was used when participants did not have a clear comprehension of the questions. All interviews were held in the language of the respondents’ choice. Most of the interviews were conducted in Dutch or French by one of the researchers. In order to achieve maximum participation of participants who did not speak those languages, an interpreter attended the interviews when necessary. The interviews were digitally recorded (audacity) with the participant’s permission, and afterwards transcribed verbatim. Regarding the interviews in the presence of an interpreter, only the answers as translated by the interpreter were transcribed. All data were anonymised and analysed according to the rules of the Belgian Privacy Commission (2004) (Law of 7 May 2004).

### Participants

The qualitative data used in this study consist of anonymised transcripts of 22 individual interviews (with a mean time of 1 h 14 m 51 s) (see Table 1 for the characteristics of the participants). In the larger D-SCOPE research, 121 older adults at risk for frailty (based on risk profiles for frailty; Dury *et al.*, 2016) were interviewed. A purposive sampling procedure was used to identify, recruit and select potentially frail, community-dwelling older adults. Five homecare organisations recruited 64 respondents from their clients and 57 respondents were recruited by snowball sampling. Based on the results of the CFAI-plus, older adults were grouped into (1) not-to-low frail, (2) low-to-medium frail and (3) medium-to-high frail, for each domain of frailty (De Roeck *et al*., 2018). The CFAI-plus was part of the quantitative questionnaire administered to the participants before conducting the qualitative interviews. Another question within the quantitative questionnaire assessed the sufficiency of care and support with a one-item question, eg, ‘On a scale from 0 to 10, to what extent do you feel that the care and support you receive is sufficient?’. The objective of the present study is to explore how frail, community-dwelling older adults experience barriers in accessing formal care and support services. Therefore, we selected the interviews of participants who were medium to highly frail according to the CFAI-plus and reported to be in need of care and support at the moment of the interview [ie, having a score lower than eight (=median of the total sample) on the question: ‘On a scale from 0 to 10, to what extent do you feel that the care and support you receive is sufficient?’). This resulted in 22 respondents.

The average age of the participants was 77.8 years (range 61–94 years). A majority of the participants were females (*n*=12). Three participants were married and 12 were widowed. Three of them had a migration background (ie, born in a different country than Belgium, Table 1).

**Table 1:** Characteristics of the participants (*n*=22)

Characteristics		Total (*n*)	%
Age	77.8 years (range 61–94 years)		
Gender	Male	10	46.5
	Female	12	54.5
Marital status	Married	3	13.6
	Never married	2	9.1
	Divorced	5	22.8
	Widowed	12	54.5
Migration background	Yes	3	13.6
Type of severe frailty	Physical	10	
	Cognitive	16	
	Psychological	10	
	Social	7	
	Environmental	6	
Number of domains severe frailty	1 domain	8	36.4
	2 domains	6	27.2
	3 domains	3	13.7
	4 domains	4	18.2
	5 domains	1	4.5

### Data analysis

In this study, we performed a thematic content analysis on the data using both deductive concept-driven coding and inductive data-driven coding (Fereday and Muir-Cochrane, 2006; Elo *et al*., 2014; Hamad *et al*., 2016). First, within the deductive approach, we used the six As of access to care as sensitising concepts (Moula, 2017), in order to test if the existing framework, that has been used in previous research several times, fits in the context of community-dwelling older adults accessing formal care and support services (Vaismoradi, Turunen and Bondas, 2013). For the deductive coding, a codebook was developed using the six As of access to care (Penchansky and Thomas, 1981; Wyszewianski, 2002; Saurman, 2016) (see above) as the main labels. Following on this, we performed the inductive coding, seeking to add dimensions to the 6As and give meaning to these labels by creating sublabels. All interviews were coded and analysed using the computer software program MAXQDA (VERBI Software, Berlin, Germany), which is a content analysis package with a good interpretive style (Kuş Saillard, 2011). The 22 transcripts were analysed by the principal researcher and coded using MAXQDA. These codes were evaluated and discussed with the co-researchers and refined until consensus was reached.

## Results

The interviews revealed a whole range of barriers concerning the access to a broad spectrum of formal care and support services for community-dwelling older people. These problems not only varied from lack of financial resources to mobility problems but also inappropriate organisation of services and lack of information. We analysed the respondents’ stories using the framework of Penchansky and Thomas (1981) as it was adapted and actualised by Wyszewianski (2002) and Saurman (2016). Also, an additional seventh barrier outside the framework was mentioned by the respondents, namely ‘ageism’. Notwithstanding the fact that several barriers were perceived by older respondents, some older adults also mentioned positive experiences regarding different aspects of access to care and support.

### Affordability

‘*Affordability* is determined by how the provider’s charges relate to the client’s ability and willingness to pay for services’ (Wyszewianski, 2002: 1441) and was mentioned by a majority of older respondents as being a very important barrier.

One of the problems respondents referred to was the combination of small pensions and the increasing cost of living with care and support needs. These small pensions impeded some of the respondents, for example from moving to a more adapted housing (a retirement flat, etc.) or to carry out the necessary modifications in their home:

*“I only became a cleaning lady after school. As a consequence, I have the minimum pension. I can’t afford to pay €700 or €800 rent with my pension of €1100. Otherwise I would already be living in Evergem (i.e. where her sister lives in social housing) for a long time. That is what impedes me” (woman, 69 years, divorced).*

Not only the price of adapted housing but also the charges for extra services within impeded older adults to move:

*“I once took information for a retirement flat, which now often has several home automation systems. They said hiring them would cost me approximately €19. I thought €19 per month, that is something I can afford. But then my son had a look at the papers and asked me if I was going to pay almost €600 per month to use these home automation systems. It seemed that the price was €19 per day and not per month. That immediately changed my opinion” (woman, 80 years, widowed).*

Conversely, a respondent also mentioned the positive results of being able to move to a social apartment last year:

*“Since I’m living here, I have to pay much less for the rent and for the heating” (man, 66 years, divorced).*

Another barrier that several respondents experienced was the price of housing modifications, especially when the government is not subsidising these. Someone stated that the impossibility of getting a subsidy for a stair lift demotivated him from applying for other financial contributions:

*“The only thing I ever asked for was a stair lift. You can have that, but the government only contributes until the age of 65. When older than 65, you need to pay for it yourself. But who needs a stair lift before the age of 65? Most of the people will only need it after the age of 65. And then they don’t contribute anymore. That doesn’t make sense” (man, 72 years, widowed).*

Respondents also mentioned their ‘lack of willingness to pay’ for a service as an obstacle. A woman mentioned in this scope that she found it difficult to use her lifelong savings for care or support services, resources she would prefer to keep for her children or grandchildren:

*“And for support, the financial side plays a role. I have savings, but I don’t like to use them. I prefer to support my children and grandchildren. A part of me says: X (respondent), you saved the money, use it. Another part of me says no” (woman, 80 years, widowed).*

A man also mentioned he did not want to buy a stair lift, because the government is not contributing, which he finds illogical. As long as that is not changing, he will keep on refusing to buy one:

“*The government is not doing enough for older people. They should do more. If they only would subsidise one tenth of the price of a stairlift, that would already be a good thing. I cannot understand that we have to pay for it all on our own” (man, 72 years, widowed).*

### Availability

‘*Availability* measures the extent to which the provider has the requisite resources, such as personnel and technology, to meet the needs of the client’ (Wyszewianski, 2002: 1441), but also refers to lack of informal care and support. Availability was also regularly mentioned as a barrier.

The lack of availability of professional care services was stated. A 61-year-old Turkish woman addressed the lack of availability of a professional caregiver to help her managing her disease, because she cannot count all the time on her informal network (both her children are very busy):

*“(Interpreter of respondent): She doesn’t receive professional care. She has to do a blood test by herself. She takes her medication by herself. She can’t go with her eldest daughter because she is busy herself with her husband and her children. From time to time she stays with her youngest daughter, but that’s a single mother” (woman, 61 years, widowed)*

Another interviewee complained about her children being around, but not willing to visit her or help her.

On the other hand, a woman mentioned that she was not in need of help at the moment, but when it becomes the case, she would have the possibility to apply for it, because there are formal care services she could rely on:

*“I don’t need any help yet. But when this would be the case, I could ask the Foyer (i.e. social housing company) to come and clean my house and my windows. There are certainly possibilities” (woman, 70 years, widowed).*

In addition to the lack of formal care, the lack of informal care was also mentioned. Regarding informal care, respondents often mentioned the lack of availability of someone in their family or social network to help them when they would become dependent, or in case of an emergency. For example, a 70-year-old widower stated how it worried him to live alone in his house:

*“The lack of having someone around me. That’s the problem. I sit here in the evening and I go to sleep. That’s OK. But what do I have to do when I can’t climb the stairs anymore? Stay downstairs? What do I have to do then? That’s what I’m thinking about” (man, 70 years, widowed).*

Someone else mentioned that she felt uncomfortable about having only one informal caregiver. She worried what to do when something would occur to the woman that was helping her a lot and was hoping to find a second informal caregiver:

*“The situation has to change. I should find someone else to help me as well as X. She also brought me to the hospital last time, she even drives my car. I should find someone else like her” (woman, 80 years, widowed).*

Also, the presence of non-family members as informal caregivers was mentioned. A man especially mentioned (in a positive sense) the presence of a friend (as informal caregiver) he could rely on for any kind of assistance:

*“She really takes good care of me. She does the shopping and always brings nice things. She even takes my bank card. I completely trust her. She also has a key to my apartment. There are moments that I can’t stand up and then she enters with the key” (man, 81 years, widowed).*

A widowed woman testified about having her neighbour around when being in need:

*“Yes, my neighbour is really close. I don’t have to call her to come around. I don’t want to call my sister, she’s too stifling” (woman, 70 years, widowed).*

### Accessibility

‘*Accessibility* refers to geographic accessibility, which is determined by how easily the client can physically reach the provider’s location’ (Wyszewianski, 2002: 1441) or how easily the provider can reach the client. In this context, some respondents quoted that accessibility of services within a feasible distance was a problem.

Some respondents reported a lack of mobility which impeded them to (physically) reach certain services.

*“My receipts for the healthcare fund have to be put in an envelope in a letter box at the Hopmarkt in Aalst (i.e. the centre of the city). So, I have to ask someone to take my notes when they go to the city. And when I need information, I have to call a central telephone number in Ghent (i.e. a city 40 km away)” (woman, 80 years, widowed).*

Besides the distance of services, respondents also considered their own mobility as important regarding accessibility of services. Several people, for instance, were concerned about losing their car or driving license as it guaranteed their independence and was needed to get to services (losing their driving license would mean losing their mobility to an important extent).

*“When I get involved in a car accident right now, they (i.e. the police) will start asking questions: sir, can you still see enough? And the insurance company, will they still give me an insurance? This scares me a lot. Because when they take my car, I have a big problem. Then I would be stuck. Even taking my wife to the doctor would be a problem” (man, 81 years, married).*

However, respondents were not only talking about geographical accessibility but also other issues concerning accessibility such as waiting lists, which seem to be long when applying for services.

*“I took information for a cleaning lady with service vouchers, but there is a waiting list of six months. I decided to let it go. It is always the same story, when you ask something, you end up on a waiting list.” (man, 72 years, widowed).*

A man told about the long waiting list for his electric mobility scooter (because of a long administrative procedure within the healthcare fund) which is interfering the physical ‘accessibility’ to providers and services:

*“I decided to apply for an electric mobility scooter so I can drive around a little more, have contact. But the application for a scooter goes through the healthcare fund, like for a wheelchair. I’m already waiting for six months now and they are still not finished.” (man, 79 years, divorced).*

Within the stories of older respondents, there were also experiences of different aspects of access interfering or relating to each other. There was the story of an older man who said that his recent moving to a social apartment (after several years) not only had a possible influence on his financial situation (ie ‘affordability’ because of a cheaper rent) but also on his physical ‘accessibility’ for care providers (in order to reach him), as well as his own mobility:

*“I live on the ground floor now, this means that the social service of the municipality enter to pick me up” (man, 66 years, widowed).*

### Adequacy (or accommodation)

‘*Adequacy (or accommodation)* reflects the extent to which the provider’s operation is organised in ways that meet the constraints and preferences of the client. Of greatest concern are hours of operation, how telephone communications are handled and the client’s ability to receive care without prior appointments’ (Wyszewianski, 2002: 1441). Respondents mentioned several inadequacies within formal care services (hospitals, formal home care) such as lack of motivation among staff.

Several older respondents found it important that formal care organisations were well organised and hired well educated and motivated staff:

*“I think that the directors or people responsible have to motivate their staff. 50% or more of the people that work over there lack motivation. Especially in the care sector, there has to be motivation. I am aware it is a special profession to wash and take care of older people” (woman, 80 years, widowed).*

A respondent mentioned in this context that he did not appreciate that the social assistant was telling him what to do. In his opinion, the social assistant was being bossy:

*“Sometimes, I have words with my social assistant. She thinks she’s the boss, but actually I’m the boss” (man, 85 years, widowed).*

Another concern often mentioned were the hours of operation (and more specifically the pace of working). This concern could reflect an organisational complaint or be focused on the individual’s professional behaviour.

*“Every 14 days, they came for 20 or 25 minutes (home carers). What can they do during that time?” (man, 78 years, widowed).*

Also, the limitations in the tasks that some formal caregivers were (legally) allowed to execute were stated as an adequacy barrier:

*“If I would have a cleaning lady, she would be very limited in what she can do. I don’t think she would be allowed to move the fridge” (woman, 69 years, divorced).*

Moreover, the (lack of) quality of services was mentioned. Older people expressed the opinion that they lacked personal contact with the professional caregiver, as contacts with formal caregivers became too quick and impersonal for them.

*“The caregiving is off less quality than before. I have the feeling we became more of a number” (woman, 89 years, never married).*

### Acceptability

‘*Acceptability* captures the extent to which the client is comfortable with the more immutable characteristics of the provider, and vice versa. These characteristics include the age, sex, social class and ethnicity of the provider (and of the client), as well as the diagnosis and type of coverage of the client’ (Wyszewianski, 2002: 1441).

Regarding ‘acceptability’, our respondents indicated that they do not always trust the care providers and consequently do not accept the care:

*“You don’t know if you can trust them. People that are doing that kind of work often never studied” (man, 81 years, married).*

An older woman especially mentioned that affordability of services was not a problem for her, but even then she did not apply for a cleaning lady because she did not trust letting someone in her house:

*“I would be able to pay for it (i.e. a cleaning lady) and it would be much easier, but all my stuff is lying here open in the house and I cannot lock things. Moreover, I heard stories about other people living alone that have bad experiences. I would not completely trust it. I have a friend in Ghent that has been robbed by house staff” (woman, 74 years, never married).*

Some specific care tasks were more difficult to accept, as they were more in the personal sphere. In this case diffidence about being washed by a professional caregiver was a concern raised by a 78-years old divorced woman:

*“In the beginning, I found it difficult to be washed. In that time, I was still in much better physical condition than now” (woman, 78 years, divorced).*

### Awareness

‘*Awareness* refers to effective communication and information strategies with relevant users (clinicians, patients, the broader community), including consideration of context and health literacy’ (Saurman, 2016: 37). Concerning the communication and information aspect, respondents mostly talked about the difficulties in getting appropriate information (about financial compensations, reductions, etc.) referring to the complex healthcare system:

*“Like financial things for example. The healthcare fund gives some compensations, you see, … There are some small compensations that I don’t receive. I don’t know how to do the necessary things, I need to find a social assistant…” (woman, 89 years, divorced).*

Also the need for health literacy in finding the appropriate information was mentioned by respondents. Some respondents mentioned that without help, they would not be able to find their way in the paperwork:

*“(Daughter of respondent): All the papers you have to send to the right place to get a small contribution. She (her mother) could never do that. That is why I am doing that for her. When the invoice of the hospital comes, she will never be able to understand that. So I am doing that for her as well. Also the papers for the insurance, it’s me who has to deal with it” (woman, 94 years, widowed).*

### Ageism

Older adults also reported experiences with ‘ageism’ (ie, stereotyping and discrimination against individuals and groups on the basis of their age) as a barrier. An older man complained about the daughter of his partner, because she (ie, the daughter) wanted to take over everything and did not believe he was still able to arrange it on his own:

*“Last year, we really had a problem with her (i.e. the daughter of his partner), she wanted to do everything (i.e. the planning of their trip, etc.). Now I said: we’ll do everything on our own, because I was sick of it. Children always think they know everything better. We can deal with it on our own” (man, 84 years, divorced).*

## Discussion

This study reports on qualitative experiences concerning access to care and support for frail community-dwelling older adults, following the framework of Penchansky and Thomas (1981) as adapted and actualised by Wyszewianski (2002) and Saurman (2016), resulting in six As of access to care and support: accessibility, affordability, availability, acceptability, adequacy (or accommodation) and awareness. The research question defined for this study was the following: which barriers do frail, community-dwelling older adults perceive to access formal care and support services?

Our study shows that this framework can be confidently applied to detect concerns of access to formal care and support for frail older adults. It brings to the foreground a very broad approach to care and support going beyond pure medical care. Moreover, perceived access barriers by Belgian users are often not related to a specific system or political level or type of service but concern the broader ‘care and support’ field. This was also a conclusion of a scientific committee that evaluated pilot projects on care and support for frail community-dwelling older adults in Belgium (ie, Protocol 3 projects, chronic care projects, a pilot project on ‘integrated broad access’).

*Affordability* of services was mentioned as an important barrier. Although Belgium is a prosperous country, pensions in Belgium are rather low compared to other EU countries. Importantly, the statutory pensions in Belgium are of the lowest of all European member states (OECD, 2011). Although research indicates that Belgian older people are ‘asset-rich but income poor’ (ie, a relatively high percentage of Belgian older people own their house) (Smetcoren, 2016), our interviews showed that the affordability of care often has to do with the concern about care support through adapted housing. In this scope, the high cost of several essential extras that have to be paid (eg, home automation systems in retirement flats, or housing adaptations like the stair elevator) clearly influence affordability. Like the majority of older people, our respondents indicated they prefer to live in their own house as long as possible (Wiles *et al*., 2011). A mentioned barrier to be able to ‘age in place’ is the high cost of housing modifications, for which the government is not contributing, or only contributing a limited percentage. This research also shows that affordability can be interconnected with *accessibility*, for example negatively when not meeting conditions applied by local governments to enter social housing, or in a positive way when moving to a cheaper adapted apartment on the ground floor made it easier for providers to physically reach the client. The interviews clearly showed that improving one barrier might have a positive impact on (an)other barrier(s) as well. We also noticed concerns about the *availability* of care and support services when older people become more dependent and in need of it, both in terms of professionals and informal carers. Recent research concluded that 3.8% of community-dwelling older adults who reported to be in need of care and support did not receive this (Fret *et al*., 2017). Respondents also indicated they lacked informal care. Despite growing policy attention, the informal care network also has its limitations (eg, children having a busy career, a daughter being a single parent). This is in line with the research of Smetcoren *et al*. (2018) in which some participants mentioned the impact of not having children, while others talked about barriers to get help from children such as distance.

Concerning *accessibility*, the respondents made clear that accessibility goes beyond geographic accessibility as it is described by Wyszewianski (2002). It also concerns for example waiting lists that limit the accessibility of services. This is in line with the research results of Bleustein *et al*. (2014) about waiting times in healthcare. Within the theme of *adequacy* (*or accommodation*), respondents complained about lack of motivation or lack of time of professional caregivers. These concerns are shared in recent research by Kilgore (2016) about home care staffing and patient satisfaction. By using the above-mentioned framework, it became clear that it is important to take into account the often-neglected individual characteristics of the client and the provider that influence *acceptability* (ie, socio-economic characteristics, trust) (Wyszewianski, 2002). Within *awareness*, the greatest concern was the complexity of finding appropriate information or the lack of *health literacy* of older adults. Although research clearly shows that Belgian healthcare performs effectively and is of good quality (Vrijens *et al*., 2015), the organisation is rather complex and shredded (especially after the sixth Belgian State Reform of 2014) (Schokkaert, 2016). It was particularly clear that the aspect of awareness influenced access to care and support for our respondents, especially for those with limited health literacy. We also discovered a seventh barrier (a seventh A) within the results, namely *ageism* which concerns stereotypes towards older adults that are described in the literature as a barrier for qualitative elderly care (Kane and Kane, 2005; Reyna *et al*., 2007).

### Limitations and future research

This study contains some limitations. First, we used interviews which were conducted not solely in the context of this paper and that have been collected to answer different research questions. In order to overcome this limitation, the quality of the data has been assessed through pre-analyses and discussion (the labels, codes and results were discussed in‐depth with the other investigators and refined through a process of consensus), and the investigators explored if the data fitted appropriately the research questions (Hox and Boeije, 2005). Second, the framework we used is an adapted and actualised version of the original framework of Penchansky and Thomas (1981) to which the aspect of awareness by Emily Saurman (2016) was added. This might be one of the first studies that has used this new framework within the context of access to care and support of frail community-dwelling older adults. Although we identified some interesting results and discovered an additional barrier within the data (ageism), further research could provide more evidence on the general applicability of this framework. Third, it would be particularly interesting to explore if any barriers were more important to those with different types of frailty, or who were frail across a greater number of domains as we focused in this paper on a general population of frail community-dwelling older adults. Future research could provide some more evidence.

## Conclusion and policy implications

Within the scope of frail community-dwelling older adults, this study brings to attention that (despite all policy measures) access to a broad spectrum of care and support services remains a challenge for our ageing society. The framework used within this research seems to be a broad and comprehensive framework. We also reported on perceived barriers that did not exactly fit within the classical definition of 1 of the 6As, but were related to 1 of the 6As. We also discovered a seventh barrier (a seventh A) within the results, namely ‘ageism’. The respondents’ barriers to access care and support go beyond solely medical services; they also involve the availability of having someone around when they are in need, waiting lists, the price of housing modifications or home automation systems, etc. This might be a challenge for our society towards enhancing policy attention to community-based care and support, where more care and support tasks are entitled to local actors (Dury, 2018; Smetcoren *et al*., 2018). Although the concept of access goes much further than affordability, the financial aspect was often mentioned, referring to a lot of Belgian older adults having limited resources and low pensions (Litwin and Sapir, 2009) and seems to remain the most important barrier within the Penchansky and Thomas framework. The aspect of affordability seems clearly *interconnected* with awareness and accessibility, referring to the complex organisation of the Belgian State and difficult procedures to get access to financial compensations. A system of automatic entitlement might give an answer to that (Moffatt and Scambler, 2008). In recent years, a project to proactively entitle a higher reimbursement status for medical care to people with low incomes already showed promising results and indicated that automatic entitlement might be an effective strategy to improve access to different kind of services (Goedemé *et al*., 2017). Another recent measure (since 2012) that provided good results was to give the possibility to low-income and vulnerable Belgian inhabitants to consult their general practitioner for one euro (the rest of the fee is paid directly to the general practitioner by the healthcare fund). It might be effective to enable other caregivers to apply this system for their patients (CM, 2018). The results also point to the complex and illogical Belgian care legislation or complex procedures, especially for older adults with limited health literacy. The impossibility of obtaining official recognition and the necessary contributions (for housing adaptations, etc.) when becoming disabled after the age of 65 is just one example. This should be a permanent point of attention for politicians to keep in mind. This paper also made clear that the framework of Penchansky and Thomas (1981) as adapted and actualised by Wyszewianski (2002) and Saurman (2016) is also applicable for detecting barriers in access to a broad range of formal care and support services (going beyond solely medical care for frail community-dwelling older adults).
